# Nasalization of Central Retinal Vessel Trunk Predicts Rapid Progression of Central Visual Field in Open-Angle Glaucoma

**DOI:** 10.1038/s41598-020-60355-1

**Published:** 2020-03-02

**Authors:** Kilhwan Shon, Youn Hye Jo, Joong Won Shin, Junki Kwon, Daun Jeong, Michael S. Kook

**Affiliations:** Department of Ophthalmology, College of Medicine, University of Ulsan, Asan Medical Center, Seoul, Korea

**Keywords:** Medical research, Outcomes research

## Abstract

Central visual field (CVF) loss is important in maintaining vision-related quality of life in eyes with open-angle glaucoma (OAG). The present study investigated whether nasalized location of central retinal vessel trunk (CRVT) at baseline is associated with rapid rate of CVF loss in early-stage OAG eyes. This study included 76 OAG eyes with high nasalization CRVT [HNL] group and 75 OAG eyes with low nasalization CRVT [LNL] group matched for glaucoma severity at baseline that showed progressive visual field (VF) loss. The rates of mean threshold changes at various regions were compared in the two groups using a linear mixed model. Clinical variables associated with rapid rate of CVF progression were also identified using a linear mixed model. The rate of CVF loss in the central 10° was significantly higher in the HNL group than that in the LNL group (−0.452 dB/year vs. −0.291 dB/year, *P* < 0.001). The average and inferior hemi-macular ganglion cell inner plexiform layer (GCIPL) progression rates were significantly faster in the HNL group than in the LNL group (*P* < 0.05). Nasalized location of CRVT was an independent predictor of a more rapid VF loss in the central 10° region (*P* < 0.05).

## Introduction

The central visual field (CVF), which includes the 12 central-most points on standard 24–2 visual field (VF) testing, is strongly associated with activities of daily living, including “reading and seeing detail”^1,2^. Hence, there would be clinical benefit in predicting CVF loss in the early stages of glaucoma, especially in eyes with progressive VF loss^2,3^. Studies indicate that CVF loss may be associated with systemic and localized vascular insufficiency of the optic nerve head (ONH) in glaucoma patients^4–7^. Central scotoma in patients with early-stage glaucoma is often associated with nocturnal hypotension, migraine, Raynaud’s phenomenon, and sleep apnea^4,5^. In addition, localized microvasculature dropout in the choroid surrounding the ONH has been linked to CVF defects in open-angle glaucoma (OAG) eyes^6,7^. Nonetheless, it is of clinical relevance to identify other ocular/systemic conditions that are associated with CVF loss in glaucoma.

The location of the central retinal vessel trunk (CRVT), which marks the exit position of the retinal vessels on the ONH, has been known to be associated with relative protection of nearest part of the neuroretinal rim tissue; the further away the region from the CRVT, the more likely it is affected by neuroretinal rim loss in glaucoma^8^. In addition, the location of the central retinal vessel trunk (CRVT), has a close association with the lamina cribrosa (LC) beam thickness and surface depth in glaucoma^9,10^. Moreover, the CRVT is located more nasally in glaucoma suspect and glaucomatous eyes compared with that in healthy eyes^11,12^. Furthermore, nasalization of the CRVT was frequently found in OAG eyes with CVF loss at initial presentation regardless of glaucoma severity^13,14^. These findings suggest that CRVT nasalization may be a potential structural clue for CVF loss in glaucoma. Therefore, one may hypothesize that OAG eyes with nasalized CRVT may exhibit enhanced susceptibility to CVF loss rather than peripheral visual field (PVF) loss during disease progression. To the best of our knowledge, however, no reports in the ophthalmic literature have evaluated whether a nasalized CRVT is associated with a more rapid VF loss at a specific location (i.e., the CVF region) over time in OAG patients.

Therefore, we have performed a longitudinal cohort analysis to compare regional VF progression rates in two early-stage OAG groups with different CRVT positions (high vs. low nasalization of CRVT) and document glaucomatous progression. Additionally, the association between various clinical variables, including CRVT location, and rapid CVF progression was assessed.

## Results

After reviewing 330 consecutive eyes in 330 OAG patients, 151 eyes of 151 OAG patients met our inclusion criteria, including 75 low nasalization CRVT (LNL) eyes and 76 high nasalization CRVT (HNL) eyes, matched for glaucoma severity (mean deviation [MD] ≥ −6 dB). There was an excellent inter-examiner agreement regarding the location of CRVT (k = 0.985). The inter-examiner correlation coefficients were 0.937 for disk tilt ratio, 0.893 for torsion degree, 0.912 for nasalization index (NI), and 0.923 for adjusted β-zone parapapillary atrophy (β-PPA) area.

**Figure 1:**
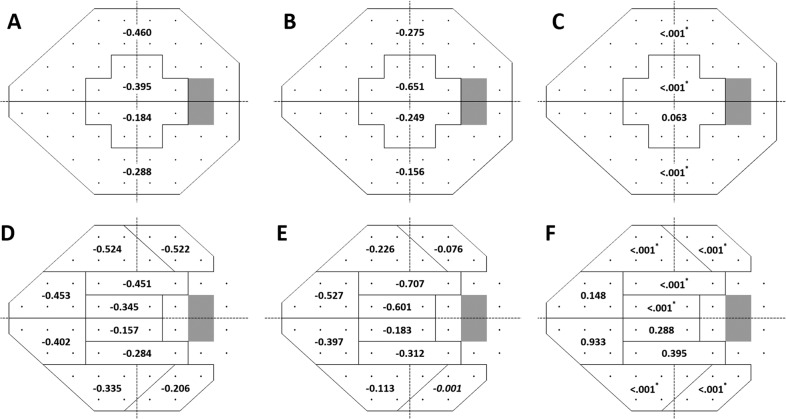
Diagrams comparing sectoral visual field loss rates of open-angle glaucoma patients with low nasalization central retinal vessel trunk (CRVT) (LNL) and high nasalization CRVT (HNL) based on a 10–24° map (**A**–**C**) and Glaucoma Hemifield Test map (**D**–**F**): (**A**), LNL group; (**B**), HNL group; (**C**) *P* values, (**D**) LNL group, (**E**) HNL group, (**F**) *P* values. *P* values are based on a linear mixed model that controlled all covariates, including follow-up period, age, gender, laterality, spherical equivalent, central corneal thickness, follow-up mean intraocular pressure (IOP), follow-up peak IOP, follow-up IOP fluctuation, and baseline mean deviation and pattern standard deviation. *Indicates statistical significance.

Compared with those in the LNL group, the eyes in the HNL group were younger (45.5 vs. 57.8 years; *P* < 0.001), more myopic (−2.86 D vs. −0.57 D; *P* < 0.001), and had higher baseline NI (0.84 vs. 0.52; *P* < 0.001), final NI (0.85 vs. 0.54; *P* < 0.001), and prevalence of β-PPA (63.1% vs. 36.0%; *P* < 0.001). Otherwise, there were no significant demographic differences between the two groups, including MD and pattern standard deviation (PSD) values at baseline and the final visits (*P* > 0.05, respectively). In the regional mean threshold (MT) values, the LNL group showed lower VF MT in the superior peripheral region, specifically in the glaucoma hemifield test (GHT)-S4 (25.1 dB vs. 26.5 dB; *P* = 0.04) and GHT-S5 (24.3 dB vs. 26.2 dB; *P* = 0.02) at baseline (Fig. 1). However, there were no significant regional differences in the MT values between the two groups in the 10–24° map (*P* > 0.05) (Table 1).

**Table 1:** Demographics and ocular characteristics of 75 open-angle glaucoma eyes with low nasalization of central retinal vessel trunk location and 76 open-angle glaucoma eyes with high nasalization of central retinal vessel trunk location.

	LNL (n = 75)	HNL (n = 76)	*P* value
Age, year	57.8 ± 11.3	45.5 ± 15.1	<0.001^*^
Sex (M/F), n	41/34	38/38	0.566
Baseline BCVA, LogMAR	0.08 ± 0.15	0.07 ± 0.16	0.715
Baseline nasalization index	0.52 ± 0.08	0.84 ± 0.08	<0.001^*^
Last F/U nasalization index	0.54 ± 0.06	0.85 ± 0.07	<0.001^*^
Spherical equivalent, D	−0.57 ± 2.39	−2.86 ± 3.17	<0.001^*^
β-PPA, n (%)	27 (36.0)	38 (63.1)	<0.001^*^
Adjusted β-PPA area^†^	0.40 ± 0.22	0.39 ± 0.25	0.712
CCT, μm	536 ± 30	536 ± 47	0.967
Tilt ratio	1.15 ± 0.4	1.18 ± 0.15	0.578
Torsion degree	2.5 ± 10.1	−0.6 ± 10.7	0.063
Baseline IOP, mmHg	14.5 ± 3.3	13.9 ± 3.2	0.259
F/U Mean IOP, mmHg	13.5 ± 2.1	13.6 ± 1.7	0.698
F/U Peak IOP, mmHg	16.4 ± 3.3	16.1 ± 2.8	0.551
F/U Fluctuation IOP	5.3 ± 3.4	4.9 ± 3.1	0.400
*A*verage glaucoma eye drops, n	1.1 ± 0.7	0.9 ± 0.8	0.232
Baseline RNFL thickness, µm	81.5 ± 7.0	82.2 ± 7.4	0.332
Last F/U RNFL thickness, µm	72.5 ± 5.9	74.2 ± 6.5	0.431
Baseline VF MD, dB	−2.5 ± 2.4	−2.3 ± 2.3	0.349
Last F/U VF MD, dB	−7.2 ± 4.1	−6.7 ± 3.8	0.154
Baseline VF PSD, dB	4.8 ± 2.9	4.2 ± 2.6	0.257
Last F/U VF PSD, dB	8.6 ± 3.3	9.3 ± 3.2	0.197
Baseline VF GHT-S1 MT, dB	29.3 ± 4.3	29.3 ± 5.1	0.936
Baseline VF GHT-S2 MT, dB	27.1 ± 4.1	27.9 ± 4.4	0.219
Baseline VF GHT-S3 MT, dB	25.2 ± 4.6	25.2 ± 5.1	0.998
Baseline VF GHT-S4 MT, dB	25.1 ± 4.1	26.5 ± 4.2	0.040^*^
Baseline VF GHT-S5 MT, dB	24.3 ± 5.2	26.2 ± 4.5	0.020^*^
Baseline VF GHT-I1 MT, dB	30.5 ± 3.0	30.5 ± 4.5	0.924
Baseline VF GHT-I2 MT, dB	29.3 ± 3.0	29.4 ± 4.3	0.825
Baseline VF GHT-I3 MT, dB	26.2 ± 4.4	26.5 ± 5.0	0.732
Baseline VF GHT-I4 MT, dB	27.5 ± 3.1	28.6 ± 4.0	0.070
Baseline VF GHT-I5 MT, dB	27.8 ± 3.3	28.7 ± 4.2	0.141
Baseline central 10° sup. MT, dB	28.7 ± 4.2	29.0 ± 4.4	0.628
Baseline central 10° inf. MT, dB	30.2 ± 2.7	30.2 ± 4.3	0.904
Baseline 10–24° sup. MT, dB	25.9 ± 3.6	26.9 ± 3.9	0.116
Baseline 10–24° inf. MT, dB	27.8 ± 2.7	28.4 ± 3.9	0.292
F/U period, years	10.0 ± 3.5	9.5 ± 3.3	0.369
Number of VF tests, n	13.7 ± 4.4	13.6 ± 4.4	0.892
Global VF rate, dB/year^‡^	−0.466	−0.442	0.668
Central 10° VF rate, dB/year^‡^	−0.291	−0.662	**<0.001**^**‡**^
10–24° VF rate, dB/year^‡^	−0.636	−0.234	**<0.001**^**‡**^
GHT-S1, S2, I1,I2 rate, dB/year^‡^	−0.249	−0.665	**<0.001**^**‡**^
GHT-S3, S4, S5, I3, I4, I5 rate, dB/year^‡^	−0.687	−0.223	**<0.001**^**‡**^

M = male; F = female; n = number; LNL = low nasalization of central retinal vessel trunk location group; HNL = high nasalization of central retinal vessel trunk location group; BCVA = best-corrected visual acuity; β-PPA = β-zone parapapillary atrophy; CCT = central corneal thickness; IOP = intraocular pressure; RNFL = retinal nerve fiber layer; VF = visual field; MD = mean deviation; PSD = pattern standard deviation; GHT = Glaucoma Hemifield Test; MT = mean threshold; F/U = follow-up.

*Statistically significant difference between LNL and HNL groups using chi-squared test for categorical data and unpaired Student’s t-test for continuous data.

^†^Average value of β-PPA area/Disc area in eyes with β-PPA.

^‡^Estimated with linear mixed model.

Values with statistical significance are shown in boldface.

The global rates of VF loss did not differ between the LNL eyes and HNL eyes (−0.360 dB/year vs. −0.310 dB/year; *P* = 0.268), after adjusting for covariates. However, in the 10–24° map, the rates of CVF loss in the central 10° were significantly higher in the HNL group than those in the LNL group (−0.452 dB/year vs. −0.291 dB/year, *P* < 0.001). In contrast, the rates of VF loss in the peripheral 10–24° regions were significantly higher in the LNL group than those in the HNL group (−0.376 dB/year vs. −0.214 dB/year, *P* < 0.001, Table 1). Similar trends are also noted with the GHT map, in which the rate of CVF (GHT-S1, -S2, -I1, and -I2) loss was significantly higher in the HNL group, whereas the rate of PVF (GHT-S3, -S4, -S5, -I3, -I4, and -I5) loss was significantly greater in the LNL group (*P* < 0.001, both, Table 1).The proportion of eyes with VF defects within the central 10° was not different between the two groups (46.1% vs. 50.7%; *P* = 0.571) at baseline. However, it was significantly higher in the HNL group compared with that in the LNL group at the last follow-up (79.5% vs. 60.3%; *P* = 0.01) (Table 2).

**Table 2:** Comparison of 75 open-angle glaucoma eyes with low nasalization of central retinal vessel trunk location and 76 open-angle glaucoma eyes with high nasalization of central retinal vessel trunk location in the frequency of visual field defects in the central 10° or the peripheral 10° to 24° regions at baseline and the last follow-up.

	LNL	HNL	*P*
**Baseline**			
Central 10°	38 (50.7%)	35 (46.1%)	0.571
Peripheral 10–24°	37 (49.3%)	41 (54.0%)	0.571
**Last follow-up**			
Central 10°	47 (60.3%)	58 (79.5%)	0.010^*^
Peripheral 10–24°	68 (87.2%)	56 (76.7%)	0.094

LNL = low nasalization of central retinal vessel trunk location group; HNL = high nasalization of central retinal vessel trunk location group.

*Statistical significance at the *P* < 0.05.

Values with statistical significance are shown in boldface.

When divided into superior and inferior central 10° and 10–24° regions, the rates of VF loss in the superior central region were significantly higher in the HNL group than those in the LNL group (−0.651 dB/year vs. −0.395 dB/year, *P* < 0.001), whereas they were marginally higher in the HNL group than those in the LNL group in the inferior central region (−0.249 dB/year vs. −0.184 dB/year, *P* = 0.063). In contrast, the rates of VF loss in the peripheral 10–24° region were significantly higher in the LNL group compared with those in the HNL group, both in the superior (−0.460 dB/year vs. −0.275 dB/year; *P* < 0.001) and inferior hemifield (−0.288 dB/year vs. −0.156 dB/year; *P* < 0.001) (Fig. 1).

**Figure 2:**
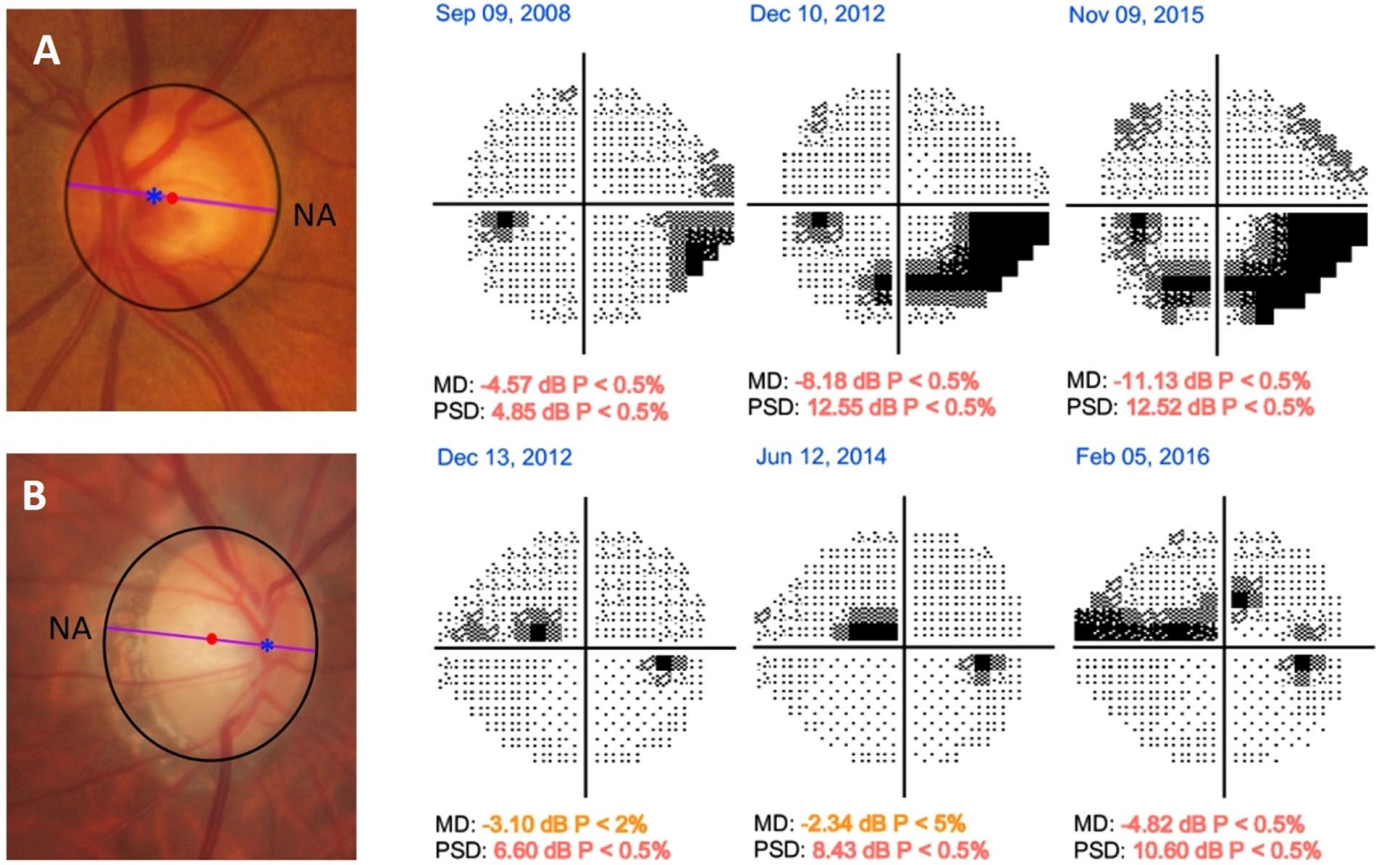
Representative case (**A**) of the left eye in a 59-year-old open-angle glaucoma (OAG) patient with a spherical equivalent (SE) of +1.25 diopter (**D**) and nasalization index (NI) of 0.63 and shift index (SI) of 0.11 that belongs to the low nasalization central retinal vessel trunk (LNL) group at baseline and shows visual field (VF) progression rate of −0.28 dB/year in the central 10° region and −0.45 dB/year in the peripheral 10° to 24° region during follow-up. Representative case (**B**) of the right eye in a 58-year-old OAG patient with an SE of −2.50 D and NI of 0.84 and SI of 0.60 that belongs to the high nasalization central retinal vessel trunk (HNL) group at baseline and shows VF progression rate of −0.88 dB/year in the central 10° region and −0.10 dB/year in the peripheral 10° to 24° region during follow-up. Blue asterisks represent the location of central retinal vessel trunk on the nasalization axis (NA). The red dots indicate the disk center based on the Bruch membrane opening margin, and the violet lines indicate the NA.

In the GHT map, the rates of VF loss were higher in the HNL group compared with those in the LNL group in the superior central region (GHT-S1; −0.601 dB/year vs. −0.345 dB/year; *P* < 0.001) and superior paracentral region (GHT-S2; −0.707 dB/year vs. −0.451 dB/year; *P* < 0.001). In contrast, LNL eyes had significantly more rapid rates of VF loss in the superior peripheral regions (GHT-S4; −0.524 dB/year vs. −0.226 dB/year; *P* < 0.001, and GHT-S5; −0.522 dB/year vs. −0.076 dB/year; *P* < 0.001) and inferior peripheral regions (GHT-I4; −0.335 dB/year vs. −0.113 dB/year; *P* < 0.001, and GHT-I5; −0.206 dB/year vs. −0.001 dB/year; *P* < 0.001). There were no significant differences in the rates of VF loss between the LNL and HNL groups in the nasal regions, both superior (GHT-S3; −0.453 dB/year vs. −0.527 dB/year; *P* = 0.148) and inferior (GHT-I3; −0.402 dB/year vs. −0.397 dB/year; *P* = 0.933) (Fig. 1). Representative cases of the two groups are shown in Fig. 2. An LNL eye shows progressive VF loss in the PVF area, whereas the initial CVF scotoma rapidly enlarges and extends into the PVF area in the HNL eye, despite having similar severity of VF loss at baseline.

Table 3 shows the results of structural progression rates based on the guided progression analysis (GPA; Carl Zeiss Meditec, Dublin, CA) software provided by the Cirrus spectral-domain optical coherence tomography (SD-OCT, Carl Zeiss Meditec). There were no significant differences in the average and superior and inferior quadrant retinal nerve fiber layer (RNFL) progression rates between the two groups. However, the average and inferior hemi-macular ganglion cell inner plexiform layer (GCIPL) progression rates were significantly faster in the HNL group than in the LNL group.

**Table 3:** Comparison of parapapillary retinal nerve fiber layer (RNFL) and macular ganglion cell inner plexiform layer (GCIPL) thickness progression rates based on the guided progression analysis (GPA) software of 75 open-angle glaucoma eyes with low nasalization of central retinal vessel trunk location and 76 open-angle glaucoma eyes with high nasalization of central retinal vessel trunk location.

Progression rates	LNL (n = 75)	HNL (n = 76)	P value
**RNFL progression rate, µm/year**			
Average	−0.75 ± 0.66	−0.53 ± 0.64	0.071
Superior quadrant	−1.15 ± 1.12	−0.82 ± 1.01	0.052
Inferior quadrant	−1.36 ± 1.26	−1.25 ± 1.21	0.741
**GCIPL progression rate, µm/year**			
Average	−0.38 ± 0.37	−0.75 ± 0.52	0.008*
Superior hemi-macula	−0.39 ± 0.41	−0.61 ± 0.52	0.087
Inferior hemi-macula	−0.35 ± 0.47	−0.92 ± 0.90	0.014*

Data are reported as mean ± standard deviation or n (%). *P < 0.05 by independent t-tests. Abbreviations: LNL, low nasalization of central retinal vessel trunk location group; HNL, high nasalization of central retinal vessel trunk location group; n, number; RNFL, retinal nerve fiber layer; GCIPL, ganglion cell inner plexiform layer.

Our linear mixed model that controlled for all covariates showed that higher NI as well as being in HNL group were significant independent predictors of more rapid VF loss in the central 10° region (*P* = 0.048, *P* < 0.001, respectively) and central GHT map region (GHT-S1 and GHT-I1, *P* = 0.048, *P* < 0.001, respectively). Other significant variables associated with faster VF loss in the central 10° and central GHT map regions included older age at baseline (*P* = 0.008 and 0.009, respectively), myopic refraction (*P* = 0.052 and 0.018, respectively), larger adjusted β-PPA area (*P* = 0.002 and 0.027, respectively), lower baseline MD (*P* = 0.029 and 0.019, respectively), and higher baseline PSD (*P* < 0.001, respectively) (Table 4).

**Table 4:** Effect of baseline covariates on slope (difference in slope, dB/year) of the central 10° region and central Glaucoma Hemifield Test map region (GHT-S1 and I1) of the entire open-angle glaucoma cohort using the linear mixed model.

Effect	Central 10°		Central (GHT-S1 and I1)	
	Estimate (95% CI)	*P*	Estimate (95% CI)	*P*
Age at baseline, per 10 years	0.04 (0.01, 0.07)	0.008^*^	0.04 (0.01, 0.08)	0.009^*^
Gender, male	−0.01 (−0.08, 0.06)	0.806	−0.05 (−0.13, 0.02)	0.172
Nasalization group, HNL	6.24 (2.11, 14.33)	<0.001^*^	6.22 (2.10, 15.28)	<0.001^*^
^Nasalization index, per 1 unit	−0.29 (−0.57, −0.00)	0.048^*^	−0.31 (−0.62, −0.00)	0.048^*^
SE, per 10 diopters	0.11 (0.00, 0.22)	0.052	0.15 (0.03, 0.27)	0.018^*^
Adjusted β-PPA area^†^	−0.11 (−0.04, −0.17)	0.002^*^	−0.08 (−0.01, −0.15)	0.027^*^
Torsion degree, per 10°	−0.01 (−0.05, 0.02)	0.536	−0.02 (−0.06, 0.02)	0.344
Disc tilt ratio, per 1 unit	−0.21 (−0.42, 0.00)	0.055	−0.32 (−0.54, −0.09)	0.006^*^
Baseline RNFLT, per 10 µm	−0.16 (−0.05, 0.32)	0.253	−0.14 (−0.07, 0.29)	0.488
Baseline MD, per 10 dB	0.18 (0.02, 0.33)	0.029^*^	0.20 (0.03, 0.37)	0.019^*^
Baseline PSD, per 10 dB	−0.29 (−0.42, −0.16)	<0.001^*^	−0.27 (−0.41, −0.12)	<0.001^*^
Mean IOP, per 10 mmHg	0.12 (−0.05, 0.29)	0.153	0.04 (−0.14, 0.22)	0.638
Peak IOP, per 10 mmHg	0.03 (−0.09, 0.15)	0.661	−0.02 (−0.15, 0.11)	0.795
Fluctuation IOP, per 10 mmHg	0.00 (−0.12, 0.13)	0.937	−0.01 (−0.14, 0.12)	0.901

GHT = glaucoma hemifield test, ^Baseline nasalization index, *Indicates statistical significance at the P < 0.05.

HNL = high nasalization central retinal vessel trunk group; SE = spherical equivalent; dB = decibel; CI = confidence interval; CCT = central corneal thickness; β-PPA = β-zone parapapillary atrophy; RNFLT = retinal nerve fiber layer thickness; MD = mean deviation; PSD = pattern standard deviation; IOP = intraocular pressure.

^†^β-PPA area/Disc area. Values with statistical significance are shown in boldface.

## Discussion

Previous cross-sectional studies have reported that nasalized CRVT is consistently associated with CVF defects in glaucomatous eyes regardless of disease severity^13,14^. The proposed explanations for this finding include both mechanical and vascular theories. CRVT may act as a stabilization support preventing glaucomatous deformation in the LC. Therefore, a nasalized CRVT may result in less mechanical support for the LC in the temporal region, which corresponds to CVF area. A nasalized CRVT may also compromise the adequacy of vascular supply in the temporal region, leading to thinning of the RNFL in the macular region and CVF loss^14^. Furthermore, the correlation between CRVT location and CVF loss was significantly stronger in moderate to severe glaucoma than that in mild glaucoma, suggesting that CRVT nasalization is not the result of glaucoma progression, but rather a stable anatomic parameter that may be a risk factor for development of CVF loss in patients with glaucoma^14^. Our findings are consistent with the speculation that there is no significant difference in the amount of CRVT NI between baseline and last follow-up measurements in both LNL and HNL groups (Table 1).

In the current study, patients in the HNL group were younger than those in the LNL group at baseline. This finding is consistent with that of a recent study that reported that normal-tension glaucoma (NTG) patients with more nasalized CRVT were younger than those with less nasalized CRVT^15^. Moreover, eyes in the HNL group were more myopic and had higher prevalence of β-PPA than those in the LNL group. The current literature regarding the association between the location of CRVT and myopia is relatively scarce, but few studies have reported findings consistent with our results. In a prospective study of myopic children, CRVT location changed with myopic elongation and the major direction of dragging or displacement was nasal^16^. Our finding was further confirmed by a cross-sectional study with myopic NTG eyes, which demonstrated that eyes with nasalized CRVT were more myopic than those with less nasalized CRVT^15^.

In our study, both LNL and HNL groups had a similar degree of CVF MT superiorly and inferiorly at baseline, whether mapped on the 10–24° map or GHT map. Furthermore, there was no significant difference in the frequency of eyes with CVF defects between the two groups at baseline. However, the proportion of CVF defects was significantly higher in HNL eyes than that in LNL eyes at last follow-up (*P* = 0.01, Table 2). This indicates that CVF progression rates were significantly higher in HNL eyes than those in LNL eyes during the course of disease. These findings may be in agreement with previous reports, which showed that the magnitude of association between CRVT nasalization and CVF depression at presentation was significant but small in mild glaucoma (MD ≥ −6 dB). However, the association was more than three-fold in moderate glaucoma (−12 dB ≤ MD < −6 dB) and almost six-fold in severe glaucoma (MD < −12 dB) compared with that in mild glaucoma^14^.

In our study, CVF progression rates were significantly more rapid in the HNL eyes than those in the LNL eyes based on both 10–24° and GHT maps (*P* < 0.001, Table 1). In the GHT hemifield analysis, although the rates of CVF progression in the HNL group were significantly more rapid in the superior central (GHT-S1) and paracentral VF (GHT-S2) regions (*P* < 0.001, respectively) than those in the LNL group, they were not significantly different in the inferior central (GHT-I1) and paracentral (GHT-I2) regions (*P* = 0.288 and *P* = 0.395, respectively). A similar trend was also seen in the 10–24° map (Fig. 2). One of the explanations for our findings is that VF defects progress more rapidly in the superior than those in the inferior hemifield of OAG eyes in the CVF area^17,18^. Cho *et al*. reported that the VF progression rate of the superior central 10° (−0.911 dB/year) was significantly more rapid than that of the inferior central 10° (−0.16 dB/year) in NTG eyes^17^. Despite differences in the study subjects and designs, our results are in agreement with these earlier findings. Another explanation is that the inferior central VF is typically affected at a later advanced stage of glaucoma, and eyes in the early stage of glaucoma were included at the time of enrollment in the current study.

In contrast, PVF progression rates were significantly more rapid in the LNL eyes than those in the HNL eyes based on both of the 10–24° map and the GHT map (−0.636 dB/year vs. −0.234 dB/year, 10–24° map; *P* < 0.001, Table 1). In the hemifield analysis, the rates of PVF progression in the LNL group were significantly more rapid in both the superior and inferior PVF regions (*P* < 0.001, respectively) based on both of the 10–24° map and the GHT map compared with those in the HNL group. One explanation for our findings is that more temporal location of the CRVT found in the LNL eyes may act as a stabilization support for papillomacular nerve fibers, preventing or minimizing CVF loss during the course of the disease. However, these eyes may be at a greater susceptibility of glaucomatous VF progression in the superior and inferior PVF areas due to lack of sufficient connective tissue support within the superior and inferior regions of the LC^19,20^. In our study, the rates of VF progression were similar in the two groups (GHT-S3 and GHT-I3; *P* = 0.14 and *P* = 0.93, respectively) in the nasal regions (Fig. 1). The explanation for this finding is that the nasal VF area is known typically to be affected first in glaucoma, which was the case in our patients with early-stage glaucoma.

In addition to VF progression, trend-based analysis was performed to compare the structural progression rates of RNFL and GCIPL in the two group. The HNL group showed significantly faster average GCIPL progression rates (−0.75 µm/year vs. −0.38 µm/year, *P* = 0.008) compared to LNL group. The rate of inferior hemi-macular GCIPL thickness loss was significantly faster in the HNL group compared to LNL group (−0.92 µm/year vs. −0.35 µm/year, *P* = 0.014), resulting in faster superior CVF progression rate in the current study. Faster rate of GCIPL thickness loss might have caused greater speed of CVF loss as seen in our HNL group as CVF is closely associated with structural integrity of GCIPL.

Another important finding in the present study was that a more nasalized CRVT as determined by NI as well as belonging to HNL group at baseline were independently associated with a greater velocity of CVF sensitivity loss, which has an important clinical implication as rapid CVF loss compromises vision–related quality of life (QOL) as measured by the National Eye Institute Visual Function Questionnaire^21^. Clinically, our findings may suggest that eyes with highly nasalized CRVT should be considered a candidate for more aggressive treatment to prevent early loss of CVF. Likewise, a poorer baseline VF as determined by MD and PSD was also a significant predictor of fast CVF progression in our OAG patients. In other words, CVF progression is more common in advanced disease than in early disease, which is in agreement with other findings that advanced stage of glaucoma is an important risk factor for VF progression^22–25^. Since advanced glaucoma often affects CVF, the rate of CVF progression correlates well with advanced glaucoma severity.

In our study population, more myopic refraction was also associated with faster CVF loss whether mapped on the 10–24° map or GHT map (*P* = 0.052 and *P* = 0.018, respectively, Table 4). During the myopic process, when the optic disk tilts temporally and becomes depressed, it may give rise to papillomacular RNFL defects due to the shearing forces across the temporal sides of the LC^26–29^, which may further increase the vulnerability to glaucomatous VF progression in the CVF area. Furthermore, the extent of myopia has been associated with faster progression in the CVF region in NTG eyes^30^. Therefore, our results suggest that myopia may also have a significant effect on the faster progression of CVF, which is independent of the effect of CRVT nasalization. Larger adjusted β-PPA area at baseline was also associated with a faster rate of CVF progression (*P* = 0.002 for the 10–24° map, and *P* = 0.027 for the GHT map). β-PPA area is closely related to the degree of myopia, where the optic disk tilts temporally during posterior globe elongation and may weaken the structural integrity of the parapapillary sclera and LC and increase the risk of glaucoma progression in the macula and CVF area^15,18^. Of interest, in myopic children with ongoing axial elongation, enlargement of β-PPA was associated with the extent and direction of vascular trunk dragging^29^, and location of β-PPA has been shown to be associated with CRVT location in adult glaucoma patients^11^.

We must acknowledge several limitations in the current study. First, our study was retrospective in design. Consequently, there was a large number of OAG patients excluded from the initial patient list during initial screening due to failure of meeting our inclusion criteria. This could have introduced selection bias. Since our patients represent two groups of Korean OAG patients with different degrees of CRVT nasalization referred to a tertiary clinic, study results from a tertiary clinic using single ethnic group may not be applicable to other races or the general population. We classified our OAG eyes into two groups (LNL vs. HNL) and measured NI based on the location of CRVT on NA. Although there is currently no universally accepted method to classify CRVT location or quantify CRVT nasalization, the method used in the current study has been validated previously^13–15^ and may minimize the subjectivity associated with manual localization or measurement of the CRVT location in the ONH. Our subjects were highly selected groups (HNL vs. LNL) of patients based on the location of the CRVT in the ONH according to the method described by Lee *et al*.^15^. Eyes with highly nasalized location of CRVT may be closely related to myopic disc as shown in our Table 1. Therefore, we have constructed a linear mixed model to assess whether the location of CRVT is significantly associated with rapid central VF progression independent of the effects of myopia, including SE, torsion degree, disk tilt ratio, and β-PPA area. Our study consistently showed that higher NI as well as being in HNL group were significant independent predictors of faster VF loss in the central 10° and central GHT map regions as noted in the Table 4. For the evaluation of CVF progression, Humphrey 24–2 VF testing was used. However, 24–2 VF testing may not detect subtle CVF progression due to distribution of large space between neighboring test spots in the central 10° area^31^. Ideally, Humphrey VF10–2 may better detect CVF progression rates as well as defects than Humphrey 24–2 VF. In the current study, we have included the subjects with visible CRVT origin in our enrollment. This could have limited our inclusion criteria to the larger discs with central cupping in which CRVT origin is not obscured by overlying neural tissue. Therefore, eyes with small discs might have been excluded from our study since they do not usually have visible CRVT origin. Finally, since our study was exploratory in the study nature, we could have used more stringent threshold for statistical significance in our multiple comparisons.

In conclusion, there are significant regional differences in VF progression rates among early-stage OAG eyes with different CRVT location. CVF loss in the 12 central-most points on 24–2 VF tests was significantly more rapid in eyes with nasalized CRVT. Our study indicates that CRVT nasalization may be an independent structural biomarker to predict rapid CVF deterioration in OAG.

## Methods

### Study participants

Our study was approved by the Institutional Review Board (IRB) of Asan Medical Center while conforming to the principles of the Declaration of Helsinki. Written informed consent was waived by our IRB as our study was retrospective study design.

The medical records of 330 consecutive patients with OAG as seen by a glaucoma specialist (M.S.K.) between March 2008 and December 2012 at the glaucoma service of Asan Medical Center were retrospectively evaluated. Initially, all patients received comprehensive ophthalmologic examination including a review of medical history, followed by measurement of best-corrected visual acuity (BCVA), slit-lamp biomicroscopy, Goldmann applanation tonometry, gonioscopy, central corneal thickness (CCT) measurement, dilated fundus examination, digital color fundus photography, red-free RNFL photography, stereoscopic optic disk photography, VF examination using the Swedish Interactive Threshold Algorithm standard 24–2 program of the Humphrey Field Analyzer (HFA, Carl Zeiss Meditec), and imaging with Cirrus HD SD-OCT.

Diagnosis of OAG was made as follows^26,28^: BCVA ≥ 20/30; normal open anterior chamber on gonioscopic examination; glaucomatous ONH with diffuse or focal neural rim thinning; a difference in the vertical cup-to-disk ratio >0.2 between eyes, not explained by differences in disk size; and disk hemorrhage or RNFL defects along with compatible glaucomatous VF loss irrespective of intraocular pressure (IOP) level. Glaucomatous VF defects met the Anderson criteria^30^. A reliable VF had to meet the following criteria: a false-positive error <15%, a false-negative error <15%, and a fixation loss <20%^30^. When patients showed glaucomatous VF defects initially, a repeat VF was performed within 2 to 4 weeks to minimize the learning effect.

The following criteria were required to be included in the current study: newly diagnosed OAG without prior treatment; age at initial presentation >18 years; VF MD ≥ −6 dB at initial presentation (for the purpose of assessing VF progression rates in early-stage OAG eyes^32^); follow-up at our clinic of at least 6 years with regular visits at 6 to 12 month intervals; availability of at least six reliable VF datasets after exclusion of the first perimetry dataset during follow-up; and documented VF progression as determined by event-based analysis. Eyes with visible CRVT origin were included while small discs without clearly visible CRVT origin and optic discs with anomalous vascular patterns with dual trunks were excluded from the study.

Other exclusion criteria included the following: a BCVA < 20/30, pathologic myopic macula, large β-PPA affecting BCVA and VF testing, lens opacities more than C2, N2, or P2 based on the lens opacities classification system III criteria during follow-up^33^. Patients with systemic diseases that could influence the VF tests or eye surgery/laser treatment (including cataract surgery) were excluded from the study. In case of unilateral disease, the affected eye was selected while the first eye with progressive VF loss was included in patients with bilateral disease that met the inclusion criteria.

### Measurement of nasalization index of CRVT location, optic disk tilt, and torsion

**Figure 3:**
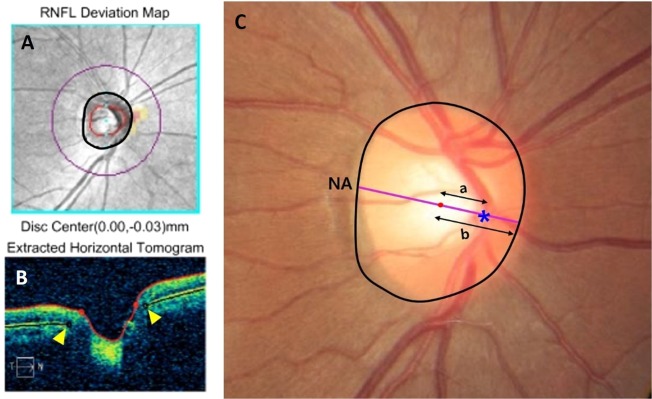
Diagrams showing measurement of nasalization index (NI), shift index (SI) and patient categorization (high nasalization central retinal vessel trunk (CRVT) [HNL] group vs. low nasalization CRVT [LNL] group) based on CRVT location in the optic nerve head (ONH). The optimally fitted ellipse around the ONH border as indicated by black circle was drawn on Cirrus HD spectral-domain optical coherence tomography (SD-OCT) retinal nerve fiber layer (RNFL) Deviation Map based on the Bruch membrane opening (BMO) margin (**A**). The disk center is automatically provided by Cirrus HD SD-OCT software as indicated by aqua blue dot. The yellow arrowheads indicate the BMO margin on the B-scan image of Cirrus HD SD-OCT (**B**). (**C**) The ellipse on Cirrus HD SD-OCT RNFL Deviation Map was overlaid onto the color fundus photography to show the optic disk margin. The location of the CRVT was demarcated on the optic disk as indicated by blue asterisk. The red dot indicates the disk center based on the BMO margin. The nasalization axis (NA) was then drawn to connect the disk center and the CRVT location from the temporal to nasal optic disk border as indicated by the violet line. NI of CRVT location was defined as the distance of the CRVT to the temporal disk border divided by the whole disk diameter on the NA. From the disk center, the distances are measured to the CRVT location (**a**) and to the nasal disk border (**b**) along the NA. If the SI (a/b) is ≥0.5, the patient/eye is categorized to have high nasalization CRVT location (HNL group). Patients/eyes with the SI < 0.5 are categorized to have low nasalization CRVT location (LNL group).

Localization of CRVT has been described previously by Wang *et al*. and Lee *et al*. (Fig. 3)^14,15^. Briefly, the optimally fitted ellipse around the ONH border was drawn on Cirrus HD SD-OCT volume scan based on the Bruch membrane opening (BMO) margin by two independent raters (K.S. and Y.H.J) who were blinded to each other’s results. The center of ellipse is automatically provided by Cirrus HD SD-OCT software. This ellipse was overlaid onto the color fundus photography to show the disk margin. The center of ellipse was to represent the disk center. The location of the CRVT was demarcated on the fundus photography without knowing the patients’ clinical information including VF results. The nasalization axis (NA) was then drawn on the fundus photography to connect the disk center and the CRVT location from the temporal to nasal disk border at the initial and last visit by the same two examiners (K.S. and Y.H.J.) using ImageJ software (version 1.52; Wayne Rasband, National Institutes of Health, Bethesda, MD). NI of CRVT location was defined as the distance of the CRVT to the temporal disk border divided by the whole disk diameter on the NA^12,13^. Average values of the NI measurements from the two examiners (K.S. and Y.H.J) were used in analyses.

Optic disk tilt ratio was defined as the ratio between major and minor axis diameter, and torsion degree was defined as the angle between the major axis and the vertical line 90° from a horizontal line connecting the disk center and fovea as described in the previous study^34^. The β-PPA area was estimated to be the total number of pixels by using the ImageJ software in a circumferential pattern^29^. The β-PPA area-to-optic disk area ratio was calculated as an adjusted β-PPA area to minimize the effect of photographic magnification error and to represent the size of β-PPA^35,36^.

### Patient grouping according To CRVT location

The study eyes were categorized into two groups on the basis of location of the CRVT in the ONH according to the method described by Lee *et al*. ^15^. From the disk center, the distances were measured to the CRVT (a) and to the nasal disk border (b) on the NA. If the shift index (a/b) is ≥0.5, the patient/eye was categorized to HNL group^15^. Eyes with the shift index <0.5 were categorized to LNL group^15^. In cases where the CRVT is located temporal to the disk center on the NA, negative value for the distance (a) was applied. Both groups were well matched in terms of glaucoma severity (MD ≥ −6 dB) according to inclusion criteria.

**Figure 4:**
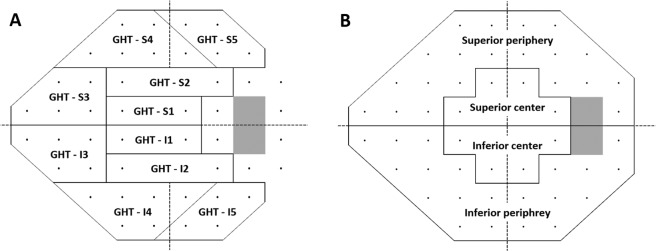
Diagrams showing 4 sectors of 10–24° map (**A**) and 10 sectors of Glaucoma Hemifield Test map (**B**).

### Outpatient follow-up

Treatment was initiated based on the following clinical factors at the time of OAG diagnosis: age, glaucoma severity, baseline and target IOP, presence of disc hemorrhage, and other risk factors. Anti-glaucoma medication was started or increased in patients already receiving treatment in the case of VF progression during follow-up in order to obtain target IOP. At last follow-up, anti-glaucoma medications included prostaglandin analogs (68.9% of patients), dorzolamide/timolol fixed combination (52.3%), topical dorzolamide (19.8%), and alpha-adrenergic agonists (29.8%). Baseline IOP was measured before treatment. The mean, peak, and fluctuation of IOP during the follow-up period were calculated.

### Location of visual field defects

We counted the VF defect clusters at baseline and the final visit, which were categorized according to their location in the central 10° or 10–24° regions based on a 10–24° map (Fig. 4)^22^. A central 10° VF defect was defined as clusters of three significant points in the central 10° with a probability <5% on the PD map or two significant points in the central 10° with a probability <1%, regardless of extension to the 10–24° VF area^22^. A peripheral 10–24° VF defect was defined as clusters in the 10–24° region without any extension into the central 10°^22^.

### Definition of visual field progression and visual field progression rates

VF progression was determined with HFA GPA (Carl Zeiss Meditec) using event-based analysis^37^. In the current study, the classification of “likely progression” was considered VF progression^37^. From each follow-up 24–2 VF test of eligible eyes, the MTs of the global, superior central 10°, inferior central 10°, peripheral superior 10–24°, and peripheral inferior 10–24° regions of the VF were collected (Fig. 1)^22^. Similarly, the MTs of the global and 10 regional clusters of the VF were also collected from the glaucoma hemifield test (GHT) map (Fig. 3)^30^. Average MTs were calculated by converting decibel values to apostilbs, averaging them, and then converting back to decibel units. VF progression rates were calculated as the changes in the average VF MT from baseline of each area of the same eye during follow-up^22,30,34,38,39^.

### RNFL and GCIPL progression analysis

Structural progression was determined with trend-based analyses for the parapapillary RNFL and macular GCIPL thickness parameters (at global, superior, and inferior regions) based on Cirrus HD SD-OCT measurement. Linear regression analysis (expressed in µm/year) was performed on the parapapillary RNFL and macular GCIPL thickness parameters using the GPA software. SD-OCT Images with poor centration, segmentation error, artifact, or a signal strength <7 were not included in the linear regression analysis.

### Statistical analyses

Inter-examiner agreements regarding the location of CRVT (HNL vs. LNL), disk tilt ratio, torsion degree, NI, and adjusted β-PPA area were assessed using Kappa statistics and intraclass correlation coefficients^26^. The two groups were compared with the t-test for continuous variables and Pearson’s chi-squared test for categorical variables. To estimate global and regional VF progression rates, a linear mixed model was used to account for confounding effects of covariates^22,34,39^. Models were fitted for fixed effects of follow-up time (years), patient age (years), gender, laterality, spherical equivalent (SE), CCT, follow-up mean IOP, follow-up peak IOP, follow-up IOP fluctuation, and baseline MD and PSD, with a random intercept for each subject^22,34,39^. For the 10 clusters of the GHT map, statistical significance was set at *P* < 0.005 for the 10 clusters of the GHT map, *P* < 0.0125 for the superior and inferior central 10° and peripheral 10–24° zones, and *P* < 0.05 for the global 24–2 area^30,34^. A linear mixed model, after adjusting for covariates, were used to compare the rates of progression for the superior and inferior central 10° and peripheral 10–24°, and the 10 GHT clusters between the LNL and HNL groups^30,34^. For structural parameters, the independent t-test was used to compare the rates of parapapillary RNFL and macular GCIPL thinning based on the GPA software between the 2 groups.

Finally, linear mixed models were constructed in all eyes to predict independent variables influencing the rate of VF progression in the central 10° region and central GHT region (i.e., GHT-S1 and GHT-I1 sector) based on age, gender, NI, SE, adjusted β-PPA area, torsion degree, disk tilt ratio, baseline MD and PSD, follow-up mean IOP, peak IOP, and IOP fluctuation^22,34^. Commercially available SAS software version 9.1.3 (SAS, Inc., Cary, NC, USA) and SPSS software version 17.0 (SPSS, Inc., Chicago, IL, USA) were used to perform all statistical analyses.
